# Genetic variation and host–parasite specificity of *Striga* resistance and tolerance in rice: the need for predictive breeding

**DOI:** 10.1111/nph.14451

**Published:** 2017-02-13

**Authors:** Jonne Rodenburg, Mamadou Cissoko, Nicholas Kayongo, Ibnou Dieng, Jenipher Bisikwa, Runyambo Irakiza, Isaac Masoka, Charles A. O. Midega, Julie D. Scholes

**Affiliations:** ^1^ Africa Rice Center (AfricaRice)01 BP 4029Abidjan 01Côte d'Ivoire; ^2^ Africa Rice Center (AfricaRice)East and Southern AfricaPO Box 33581Dar es SalaamTanzania; ^3^ Department of Animal and Plant SciencesUniversity of SheffieldSheffieldS10 2TNUK; ^4^ School of Agricultural SciencesMakerere UniversityPO Box 7062KampalaUganda; ^5^ Africa Rice Center (AfricaRice)01 BP 2551Bouaké 01Côte d'Ivoire; ^6^ Department of Plant SciencesKenyatta UniversityPO Box 43844‐00100NairobiKenya; ^7^ International Centre of Insect Physiology and Ecology (ICIPE)PO Box 30Mbita40305Kenya

**Keywords:** grain yield, *Oryza glaberrima*, *Oryza sativa*, photosynthesis, post‐attachment resistance, predictive breeding, witchweed

## Abstract

- The parasitic weeds *Striga asiatica* and *Striga hermonthica* cause devastating yield losses to upland rice in Africa. Little is known about genetic variation in host resistance and tolerance across rice genotypes, in relation to virulence differences across *Striga* species and ecotypes.
- Diverse rice genotypes were phenotyped for the above traits in *S. asiatica‐* (Tanzania) and *S. hermonthica‐*infested fields (Kenya and Uganda) and under controlled conditions.
- New rice genotypes with either ecotype‐specific or broad‐spectrum resistance were identified. Resistance identified in the field was confirmed under controlled conditions, providing evidence that resistance was largely genetically determined. *Striga*‐resistant genotypes contributed to yield security under *Striga*‐infested conditions, although grain yield was also determined by the genotype‐specific yield potential and tolerance. Tolerance, the physiological mechanism mitigating *Striga* effects on host growth and physiology, was unrelated to resistance, implying that any combination of high, medium or low levels of these traits can be found across rice genotypes.
- *Striga* virulence varies across species and ecotypes. The extent of *Striga*‐induced host damage results from the interaction between parasite virulence and genetically determined levels of host–plant resistance and tolerance. These novel findings support the need for predictive breeding strategies based on knowledge of host resistance and parasite virulence.

## Introduction

Species of the *Striga* genus (Orobanchaceae family) are obligate hemi‐parasitic plants that parasitize roots of host plants via a specialized organ called the haustorium (Musselman, 1980). *Striga* spp. (henceforward referred to as *Striga*) are most prevalent in tropical Africa, where they pose serious threats as weeds in rain‐fed cereal production systems (Parker, 2012). The most important species in rice are *Striga asiatica* and *Striga hermonthica* (Rodenburg *et al*., 2010). Together with the related but less widespread *Striga aspera* (Willd.) Benth., they constrain rain‐fed rice production in 38 African countries, with an estimated incidence rate of 12% (Rodenburg *et al*., 2016). Average *Striga*‐inflicted yield losses of rice in farmers’ fields range between 21% and 80% (Elliot *et al*., 1993; N'Cho, 2014). The extent of these losses is a function of many factors, including the *Striga* infestation level, environmental conditions and the genetic interaction between host‐plant genotype and parasite ecotype (host–parasite specificity), which determines the level of *Striga* resistance and tolerance.

Host‐plant resistance to *Striga* is defined as the ability to reduce or prevent infection (Shew & Shew, 1994), while tolerance refers to the extent to which effects of infection on the host plant are mitigated (Caldwell *et al*., 1958). Mechanisms that prevent or reduce *Striga* seed germination rates are categorized as pre‐attachment resistance, while those that prevent or reduce the success of root penetration or establishment of the vascular connection between host and parasite are called post‐attachment resistance (Yoder & Scholes, 2010). As a consequence of the large genetic variation within and between *Striga* ecotypes (populations), complete host‐plant resistance (immunity) against this parasite is rare. As host damage can be inflicted by a few parasitic infections, varieties with partial *Striga* resistance should also have good levels of tolerance to avoid yield losses in the field (Rodenburg & Bastiaans, 2011).

A number of studies have shown the existence of genetic variation in resistance to different ecotypes of *Striga* across a range of rice genotypes. For example, Harahap *et al*. (1993) showed that four genotypes of *Oryza sativa* were partially resistant to *S. hermonthica* in western Kenya. Riches *et al*. (1996) and Johnson *et al*. (1997), identified five genotypes of the African rice species *Oryza glaberrima* and two *O. sativa* genotypes with partial resistance against an ecotype of *S. aspera* and *S. hermonthica* in northern Côte d'Ivoire. Under controlled environments, Jamil *et al*. (2011) identified a number of interspecific New Rice for Africa (NERICA) cultivars with pre‐attachment resistance against *S. hermonthica*, while Cissoko *et al*. (2011) identified cultivars with post‐attachment resistance (within the same germplasm pool) against an ecotype of *S. hermonthica* and *S. asiatica*.

These studies assessed either resistance among a relatively diverse group of rice genotypes against one *Striga* species or ecotype in the field (Harahap *et al*., 1993; Jamil *et al*., 2012), or resistence among a genetically related group of rice genotypes (i.e. the NERICAs) against different *Striga* species or ecotypes (Cissoko *et al*., 2011; Jamil *et al*., 2011; Rodenburg *et al*., 2015) under controlled environment conditions. Thus, these and other studies have resulted in a pool of known genotypes resistant to a specific ecotype of *Striga*, but limited information on how broad‐spectrum resistance is against genetically different species and ecotypes, and how expression of resistance is affected by environmental variability. Moreover, none of the previous studies has conclusively established genetic variation in *Striga* tolerance in rice and the potential mechanistic background of tolerance in infected hosts. Identification of genetic sources of broad‐spectrum resistance and effective tolerance in rice germplasm is critical for marker‐assisted and conventional breeding programmes to develop useful cultivars for affected rice farmers.

The objectives of this study were therefore to determine whether *Striga* resistance among a diverse set of rice genotypes (some with previously identified resistance to *Striga*) is specific to a particular *Striga* species or ecotype, or broad‐spectrum; whether resistance against *Striga* is sufficient to maintain high rice grain yields under *Striga*‐infested conditions in different environments; and whether genetic variation in *Striga* tolerance exists in rice and through which host‐plant morphological or physiological traits this can be assessed. Achievement of the last objective would also shed light on the mechanisms underlying tolerance to this parasite.

## Materials and Methods

### Field screening trials

Twenty rice genotypes of different species and origins (Table 1), 19 of which had putative *Striga* resistance, were grown in *Striga*‐infested plots at Kyela, Tanzania (*Striga asiatica* (L.) Kuntze), and at Namutumba, Uganda, and Mbita, Kenya (both *Striga hermonthica* Benth.). The interspecific rice cultivar NERICA‐2 was included as a *Striga*‐resistant and high‐yielding check against which the performances of all other genotypes were compared across sites, *Striga* ecotypes and years. The cultivar IAC165 (*Oryza sativa* ssp. *japonica*), originally from Brazil, was included as a *Striga*‐susceptible check. Seeds of all rice genotypes were obtained from the Africa Rice Center (AfricaRice), Cotonou, Benin. Seeds of *S. asiatica* and *S. hermonthica* were collected in the previous season from plants parasitizing rice at Kyela, Tanzania (Sa‐Kyela) and Namutumba, Uganda (Sh‐Namutumba) and maize (*Zea mays* L.) at Mbita, Kenya (Sh‐Mbita) in farmers’ fields surrounding the experimental field sites. These seeds were used to supplement the existing soil seed bank in the field trials as well as for the controlled environment studies.

**Table 1:** Upland rice genotypes used in the study; their full names, species, origins, presumed reaction types to *Striga* and literature sources

Genotypes	Species and origin	Reaction type	Source
ACC102196	*Oryza glaberrima* (Liberia)	R	1 ^,^ 2
Agee	*O. glaberrima* (Ghana)	R	3
Anakila	*O. glaberrima* (Mali)	R	3
CG14	*O. glaberrima* (Senegal)	R	4 ^,^ 5 ^,^ 6 ^,^ 7 ^,^ 8
Makassa	*O. glaberrima* (Sierra Leone)	R	1 ^,^ 2
MG12	*O. glaberrima* (Mali)	R	2
Ble Chai	*Oryza sativa* ssp. indica (Thailand)	R	9
IAC165	*O. sativa* ssp. indica (Brazil)	S	3 ^,^ 4 ^,^ 5 ^,^ 6
**IR49255‐**B‐B‐5‐2	*O. sativa* ssp. indica (Philippines)	R	2 ^,^ 6 ^,^ 9
**IR38547‐**B‐B‐7‐2‐2	*O. sativa* ssp. indica (Philippines)	R	9
**UPR**‐103‐80‐1‐2	*O. sativa* (origin unknown)	R	9
WAB56‐50	*O. sativa* ssp. *japonica* (Côte d'Ivoire)	I	4 ^,^ 6 ^,^ 8
WAB56‐104	*O. sativa* ssp. *japonica* (Côte d'Ivoire)	I	4 ^,^ 5 ^,^ 6 ^,^ 8
**WAB928**‐22‐2‐A‐A‐B	*O. sativa*, ssp. *japonica* × *indica* (Côte d'Ivoire)	R	10
**SCRID090**‐60‐1‐1‐2‐4	*O. sativa* ssp. *japonica*, cv FOFIFA161 × interspecific cv NERICA‐3 (Madagascar)	R	11
**WAB935**‐5‐A‐2‐A‐A‐B	Interspecific, cv IR47 × cv CG20 (Côte d'Ivoire)	R	10
**WAB880**‐1‐32‐1‐1‐P2‐HB‐1‐1‐2‐2	Interspecific, cv WAB56‐50 × cv CG14 (Côte d'Ivoire)	R	11
NERICA‐2*	Interspecific, cv WAB56‐104 × cv CG14 (Côte d'Ivoire)	R	4 ^,^ 5 ^,^ 6 ^,^ 8
NERICA‐4	Interspecific, cv WAB56‐104 × cv CG14 (Côte d'Ivoire)	R	4 ^,^ 5 ^,^ 6 ^,^ 8
NERICA‐10	Interspecific, cv WAB56‐104 × cv CG14 (Côte d'Ivoire)	R	4 ^,^ 5 ^,^ 6 ^,^ 8

Text in bold indicates how genotype names are abbreviated.

^1^Riches *et al*. (1996); ^2^Johnson *et al*. (1997); ^3^Jamil *et al*. (2012); ^4^Cissoko *et al*. (2011); ^5^Jamil *et al*. (2011); ^6^Rodenburg *et al*. (2015); ^7^Kaewchumnong & Price (2008); ^8^Samejima *et al*. (2016); ^9^Harahap *et al*. (1993); ^10^C. Riches, NRI (pers. comm.); ^11^L. M. Raboin, Cirad (pers. comm.); R, Resistant; I, Intermediate; S, Susceptible; all interspecifics are offspring of crosses between *Oryza glaberrima* and *O. sativa* ssp. *japonica*. *NERICA‐2 is the *Striga*‐resistant and high‐yielding check.

**Figure 1:**
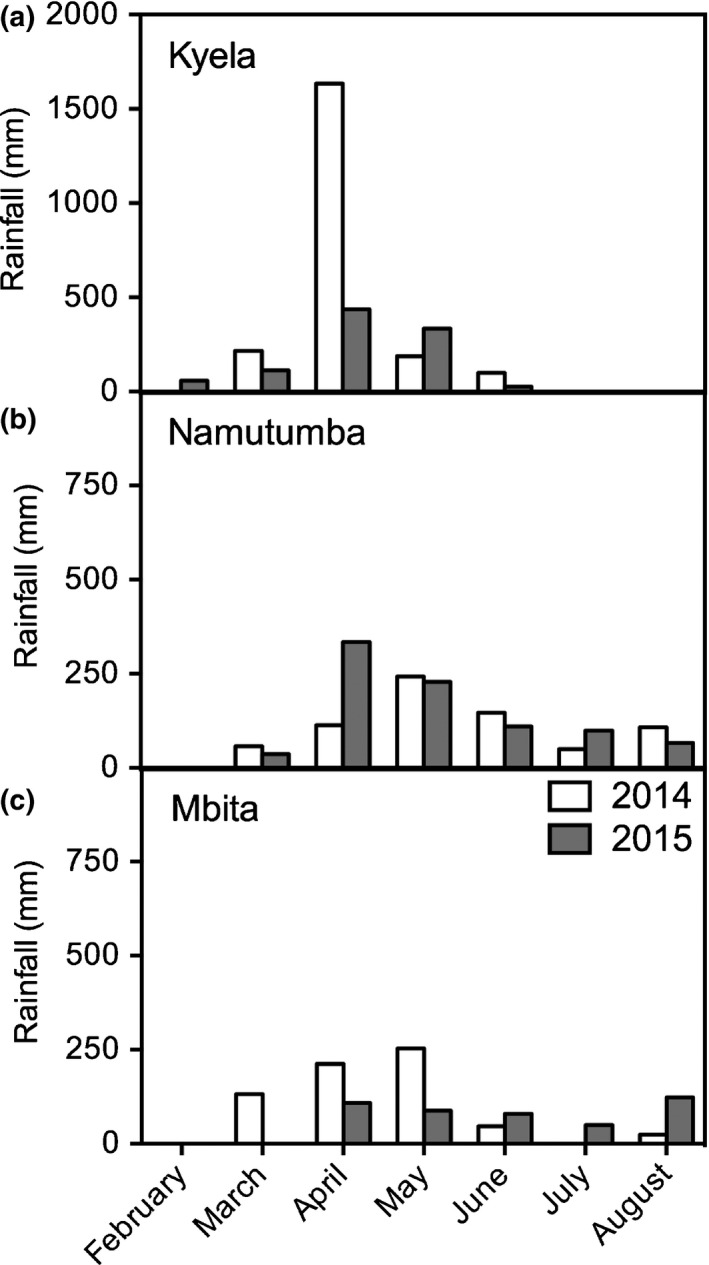
Rainfall data for field sites in (a) Kyela (Tanzania), (b) Namutumba (Uganda) and (c) Mbita (Kenya) in 2014 and 2015.

The *S. asiatica* field screening trials were conducted during the rainy seasons (February/March–July) of 2014 and 2015 in Mbako (9°35ʹS, 33°48ʹE; 525 m above sea level, asl), in Kyela District, Mbeya Region in southern Tanzania (Supporting Information Table S1). Kyela District is a *S. asiatica*‐endemic upland rice‐growing area. This screening trial was executed in an already infested farmer's field. Rainfall data were obtained from a rain gauge installed in the middle of the field (Table S1; Fig. 1).

The *S. hermonthica* field screening trials were conducted during the long rainy seasons of 2014 and 2015 (March–August/September) at two locations: in a farmer's field in Nsinze, Namutumba District, Uganda (00°51ʹN, 33°41ʹE; 1125 m asl); and at the farm of the International Centre of Insect Physiology and Ecology (ICIPE) at Mbita (0°43ʹS, 34°20ʹE; 1141 m asl), in Suba District, western Kenya (Table S1). Both trials were laid out on heavily *Striga*‐infested fields. Rainfall data in Namutumba were obtained from a nearby meteorological station, and in Mbita from ICIPE's meteorological station at the experimental farm (Table S1; Fig. 1).

All field trials were laid out in a randomized block design with six replicates. At Kyela each plot, representing an individual genotype, measured 1.25 × 3.75 m (4.69 m^2^) and contained five rows of 15 hills with an inter‐hill distance of 0.25 × 0.25 m (Table S1). At Mbita and Namutumba, each plot measured 1.25 × 2.75 m (3.44 m^2^) with five rows of 11 hills with the same hill and row distances as in Kyela. Plots were separated by one open row of 0.25 m to avoid neighbour effects. Each replicate was separated by a 1.25‐m alley.

Each plot received supplementary *Striga* seeds that were mixed with 200 g of white sand and incorporated in the upper 5–10 cm of soil. An amount of 0.21 g of *S. asiatica* seed m^−2^ (germination rate: 70%) was provided at Kyela, in both years. At Namutumba, the *S. hermonthica* seed infestation rate was 0.29 g m^−2^ in 2014 and 0.26 g m^−2^ in 2015 (germination rate 90%) and at Mbita this was 0.29 g m^−2^ (germination rate 90%) in both years.

For crop establishment and weed control, we followed procedures described in Rodenburg *et al*. (2015). At all sites, fertilizer was applied at 35 d after sowing (DAS). In Kyela, nitrogen−phosphorus−potassium (N‐P‐K; 20 : 10 : 10) was applied at an equivalent rate of 100 kg ha^−1^, while at Namutumba and Mbita, N‐P‐K (17 : 17 : 17) was applied at a rate of 50 kg ha^−1^ (Table S1).

The number of *Striga* plants that emerged within the central area of each plot (comprising 27 rice hills) was recorded regularly. At Kyela, counting was carried out at 71 and 88 DAS and at harvest in 2014, and at 43, 54, 71, 82 and 105 DAS and at harvest in 2015. In Namutumba and Mbita, *Striga* plants were counted bi‐weekly, at 43, 57, 71, 85, 99/100 DAS and at harvest in both years. These data enabled the assessment of the maximum number of emerged *Striga* plants (*NS*_max_), a measure of *Striga* resistance in the field (Rodenburg *et al*., 2005). At harvest, emerged *Striga* plants within each observation area of 27 hills in each plot were collected, oven‐dried at 70°C for 48 h, and weighed on digital weighing scales, for the assessment of *Striga* biomass dry weight.

At harvest, the height of the rice plants growing in the central nine hills was measured from ground level to the tip of the tallest panicle. Rice panicles were harvested from the same central 27 hills of each plot and air‐dried for 2 wk, after which rice grains were separated from the panicles and weighed. Grain moisture content was assessed, using a digital grain moisture meter (Model SS‐7; Satake Eng. Co., Tokyo, Japan), to correct rice grain dry weights to 14% moisture content. Rice straw biomass dry weights were assessed for plants from the central nine hills within each 27‐hill harvest area, and included all aboveground rice biomass, except panicles. The straw was oven‐dried at 70°C for 48 h before weighing.

### Resistance ranking of rice genotypes under controlled environmental conditions

To determine the impact of the field environment on the resistance ranking of the genotypes, a subset of 11 genotypes were phenotyped for post‐attachment resistance under semi‐controlled environment conditions at a screen house at AfricaRice in Dar es Salaam, Tanzania (with the Kyela ecotype of *S. asiatica* – Sa‐Kyela – and the Mbita ecotype of *S. hermonthica* – Sh‐Mbita) and fully controlled environment conditions in a screening facility at the University of Sheffield (with the Namutumba ecotype of *S. hermonthica* – Sh‐Namutumba) with parasite seeds collected from the field sites. *Striga*‐infected rice plants of each genotype were grown in a rhizotron system as described by Cissoko *et al*. (2011). Four replicates were evaluated for each genotype × *Striga* spp. combination. The genotypes tested were NERICA‐2, NERICA‐4, WAB928, WAB880, WAB56‐50, WAB56‐104, IR38547, Ble Chai, SCRID090, CG14 and the susceptible check IAC165. Ble Chai was missing in the rhizotron screen against *S. asiatica* because of germination failure. Quantification of post‐attachment resistance levels was based on total number of attachments and mean parasite biomass dry weight (assessed after oven‐drying at 50°C for 48 h) per host root system for each genotype at 21 d after inoculation (DAI).

### Determining the tolerance levels of the rice genotypes

A pot experiment was carried out in the screen house of AfricaRice, from October 2015 until February 2016, using natural incoming light (70% of light intensity outside the screen house). Plastic 10‐l pots (height: 27.5 cm; diameter: 25 cm) were filled with a sand : soil mixture at a ratio of 2 : 1. The soil was collected from the experimental farm of Sokoine University of Agriculture, in Morogoro, and the sand was collected from the shores of the Ruvu River, adjacent to the Ruvu irrigated rice scheme. This mixture contained 0.17% N, 6.7 ppm P and 228 ppm K, and had a pH (H_2_O) of 6.8 (Crop Nutrition Laboratory Services Ltd, Nairobi, Kenya). The pot experiment comprised two *Striga* levels (*Striga*‐infested and *Striga*‐free) and nine rice genotypes, following a randomized complete block design with four replications. It included the *O. glaberrima* genotypes ACC102196, CG14 and Makassa, the *O. sativa* genotypes IR38547, WAB56‐104, WAB928 and IAC165, and the interspecific genotypes WAB935 and NERICA‐10.

Thirty‐six pots (half of the experiment) were infested with *S. asiatica* seeds, and the other half contained *Striga*‐free soil (control treatments). For the *Striga*‐infestation treatment, the upper 10 cm of soil was mixed with 0.050 g of viable *S*. *asiatica* seeds. During the 10 d after *Striga* infestation, the soil in each pot was kept between field capacity and saturation to allow *Striga* seed preconditioning. Fertilizer was applied at a rate equivalent to 100 kg of N‐P‐K (17 : 17 : 17) ha^−1^ (*c*. 1.2 g per pot), and mixed with the upper 10 cm of soil during *Striga* infestation. Rice was sown at a rate of six seeds per pot (10 d after *Striga* infestation) and thinned to three plants per pot at 14 DAS. Throughout the experiment, in all pots soil moisture levels were maintained between field capacity and saturation.

Rice plant height from ground level to the tip of the tallest leaf (at 43 and 57 DAS) or panicle (at maturity) was measured to assess maximum height. At maturity, rice grains obtained from the three plants in each pot were threshed, air‐dried for 10 d and weighed. The grain moisture content of each sample was assessed to standardize grain weights to 14% moisture content. At harvest, rice straw (leaf, stem and rachis) was collected from each pot, oven‐dried and weighed to establish total aboveground straw biomass dry weight. Emerged *Striga* plants were counted every 3 d starting after the first *Striga* emergence in each pot, to assess maximum aboveground *Striga* numbers (*NS*_max_).

Photosynthesis was measured with the Li‐Cor 6400XT from Li‐Cor Bioscience (Lincoln, NE, USA). Light‐saturated leaf CO_2_ assimilation rates (*A*_max_) of rice were measured at 1200 μmol m^−2^ s^−1^ (photosynthetically active radiation (PAR); over the waveband 400–700 nm) at *c*. 30, 45 and 60 DAS (± 2 d). On each occasion, measurements were conducted on four consecutive days, with one full replicate per day, between 11:00 and 15:00 h. The same plants were used for repeated measurements. Measurements were always made halfway along the length of the youngest fully expanded leaf. During the measurements, leaf temperature ranged between 29.1 and 39.6°C. Relative humidity in the leaf chamber was controlled to stay within the range of 35–50%. The inlet CO_2_ concentration was set at 400 ppm and depletion never exceeded 20 ppm.

### Statistical analyses

Before analyses, data were checked for homoscedasticity and normality following Sokal & Rohlf (1995). Following these tests, field data on rice grain and *Striga* dry weights were analysed using a linear mixed model. We tested whether there was a significant location × year × genotype interaction effect, and, where this was the case, we fitted a model for each location (Kyela, Mbita and Namutumba) separately, and tested whether there was a significant year × genotype interaction effect within each location. We first performed a log‐likelihood ratio test for the homogeneity of variance and, when the variance was not constant, we took into account the heterogeneity of the variances. When the year × genotype interaction effect was significant (*P *<* *0.05), we fitted a model for each year separately, where genotype was considered a fixed effect and block, nested in replicate, and replicate were considered random effects. For parameters for which there was a significant cultivar effect, Dunnett's method (Dunnett, 1955) was used to compare each genotype with NERICA‐2, which was used as a control. For analyses of the maximum number of emerged *Striga* plants (*NS*_max_), a generalized linear mixed model (McCullagh & Nelder, 1989) was used under the assumption of a Poisson distribution. Least‐squares means (LS‐Means) and associated SE derived from the linear mixed model were computed. Spearman rank correlations for parameters measured in the field were calculated between LS‐Means of *NS*_max_ and *Striga* dry weight (DW_*Striga*_), between *NS*_max_ and rice grain yield, and between rice grain yield and rice plant height.

The rhizotron and pot data were analysed following checks for homoscedasticity and normality. ANOVAs were followed by a comparison of means using Tukey's honest significant difference test. *Striga*‐inflicted losses in plant height (*Height*) and light‐saturated photosynthesis (*A*_max_) were calculated relative to the *Striga*‐free control for each genotype as

$$[\left(X_{c}-X_{s,i}\right)/X_{c}]\times 100\%$$

(*X*_c_, the mean *Striga*‐free control value of parameter *X*, calculated over four replicates; *X*_*s*,*i*_, the value of parameter *X* of a *Striga*‐infected plant of replicate *i*.) The *Striga*‐inflicted losses data were analysed using a generalized linear mixed model with a binomial distribution. All data were analysed using Sas/stat software, Version 9.2 of the Sas System for Windows (SAS Institute, 2011).

## Results

### Resistance levels among diverse rice genotypes exposed to different *Striga* species and ecotypes

**Figure 2:**
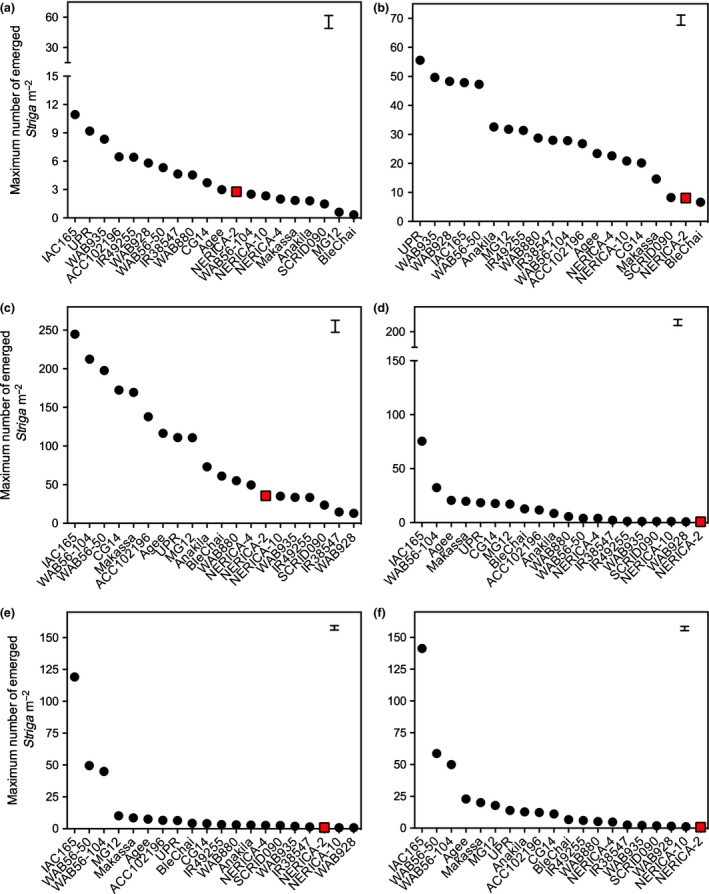
Maximum number of emerged *Striga* plants m^−2^ per rice cultivar for field trials at Kyela, Tanzania under *Striga asiatica* infestation in Kyela in (a) 2014 and (b) 2015, *Striga hermonthica* infestation in Mbita, Kenya in (c) 2014 and (d) 2015 and *S. hermonthica* infestation in Namutumba, Uganda in (e) 2014 and (f) 2015. Bars represent ± SE of the least squares (LS) means. Red boxes indicate the position of resistant check genotype NERICA‐2.

Year by rice genotype interaction effects on *NS*_max_ were highly significant (*P *<* *0.001) at all sites, requiring analysis per year (Table S2). At all sites and in all years, rice genotype had a highly significant (*P *<* *0.001) effect on *NS*_max_ (Table S2). The genotype ranking showed that the resistant check NERICA‐2 was always among the most resistant genotypes (Fig. 2). Highly significant (*P *<* *0.001) correlations were found between *NS*_max_ and *Striga* biomass in all field trials (Table S3).

In Kyela in 2015, a year with generally high *S. asiatica* infection levels, only SCRID090 and Ble Chai showed similar levels of resistance to NERICA‐2. All other genotypes had significantly (*P *<* *0.01) higher infection levels. Twelve genotypes proved moderately resistant, with Makassa, CG14, NERICA‐10, NERICA‐4 and Agee being the most resistant. Five genotypes were clearly susceptible: UPR, WAB935, WAB928, IAC165 and WAB56‐50 (Fig. 2b). In 2014, a year with generally lower infection levels in Kyela, the genotype ranking was similar, although 11 genotypes showed resistance levels equivalent to that of NERICA‐2, and two genotypes, MG12 and Ble Chai, were significantly (*P *<* *0.01) more resistant. Six genotypes (UPR, WAB935, WAB928, IAC165, IR49255 and ACC102196) were significantly (*P *<* *0.05) more susceptible (Fig. 2a).

In Mbita in 2014, a year with generally high infection levels, three genotypes, NERICA‐10, WAB935 and IR49255, had similar resistance levels to *S. hermonthica* to NERICA‐2, and three genotypes, SCRID090, IR38547 and WAB928, were significantly (*P *<* *0.01) more resistant. Thirteen genotypes had significantly (*P *<* *0.01) higher infection levels than NERICA‐2. Four of them (NERICA‐4, WAB880, Ble Chai and Anakila) proved moderately resistant, while five (IAC165, WAB56‐104, WAB56‐50, CG14 and Makassa) were very susceptible (Fig. 2c). The ranking in 2015, with generally lower *S. hermonthica* infection levels, was similar but showed less differentiation between genotypes. Five genotypes (NERICA‐10, WAB935, IR49255, SCRID090 and WAB928) had similar infection levels to NERICA‐2, and the remaining genotypes were all significantly (*P *<* *0.05) less resistant (Fig. 2d). In Namutumba, the years were more similar in terms of *S. hermonthica* infection level (Fig. 2e,f). In 2014 only five genotypes had similar resistance levels to NERICA‐2. In 2015, four of them, WAB935, SCRID090, WAB928 and NERICA‐10, were as resistant as NERICA‐2. All other genotypes were more susceptible (Fig. 2e).

**Figure 3:**
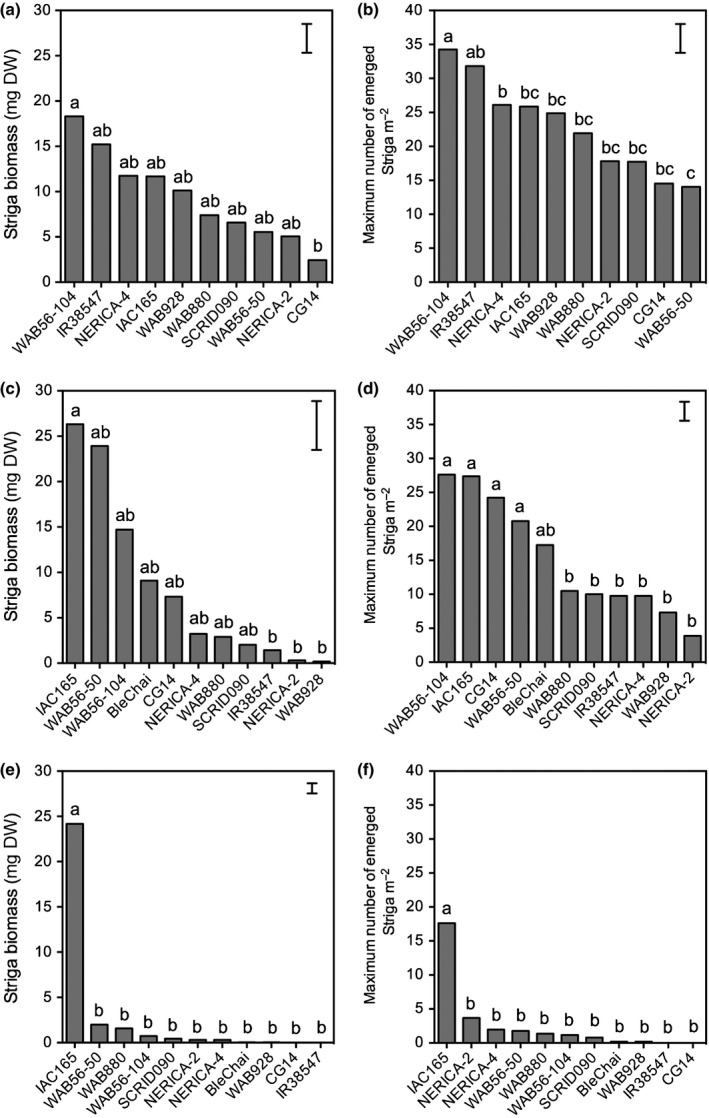
*Striga* numbers and biomass on a subset of rice genotypes observed in the rhizotron system infected with either (a, b) *Striga asiatica* from Kyela (Sa‐Ky), (c, d) *Striga hermonthica* from Mbita (Sh‐Mb) or (e, f) *Striga hermonthica* from Namutumba (Sh‐Na). Bars represent ± SE of the least squares (LS) means. Bars with different lowercase letters are significantly different (*P *<* *0.05).

**Figure 4:**
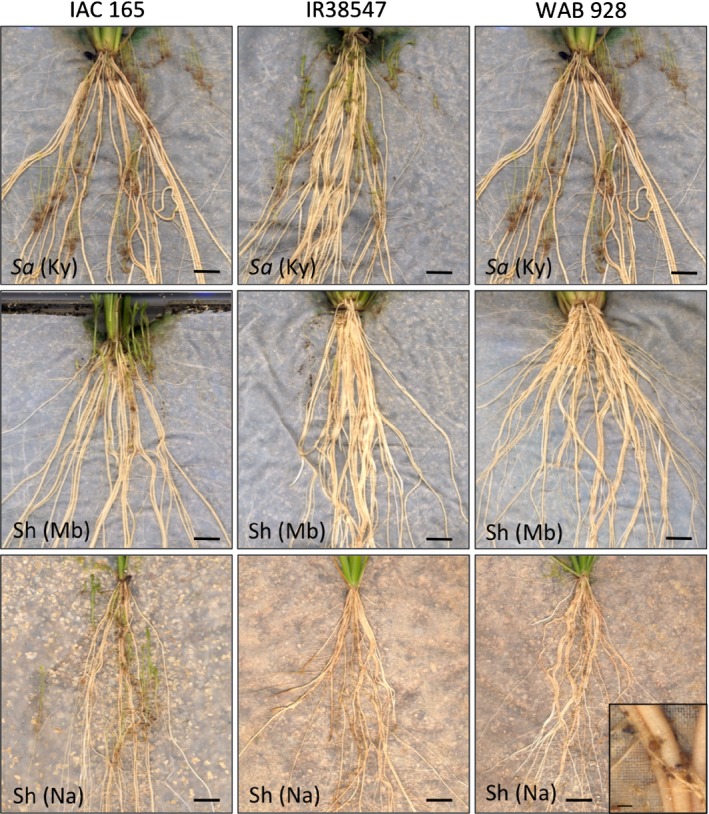
Resistance phenotypes of WAB935, WAB928, IR38547 and IAC165, screened in the rhizotron systems infected with either *Striga asiatica* from Kyela (Sa‐Ky), *Striga hermonthica* from Mbita (Sh‐Mb) or *Striga hermonthica* from Namutumba (Sh‐Na). Bars: main images, 0.5 cm; inset image, 500 μm.

In rhizotron screens (controlled environments), genotype rankings on *Striga* numbers and *Striga* biomass dry weight were similar to those observed in the field (Figs 2, 3). In both field and rhizotron screens, WAB928 showed susceptibility to *S. asiatica* (Kyela), but high resistance against both *S. hermonthica* ecotypes (Mbita and Namutumba) (Figs 3, 4). Similarly, although less pronounced, IR38547 was generally susceptible to *S. asiatica* (Kyela) in both rhizotron and field screening but resistant to both ecotypes of *S. hermonthica* (Figs 3, 4).

### Genotype‐specific crop yields across environments, *Striga* species and ecotypes

At each site, significant year by genotype interaction effects on rice grain yields under *Striga*‐infested conditions were observed (Table S4), requiring analyses per year. In each year and at each site, highly significant (*P *<* *0.0001) genotype effects on rice grain yields under *Striga*‐infested conditions were observed. Similarly, significant correlations between rice yield and rice plant height were observed in Kyela and Namutumba (Table S3).

**Figure 5:**
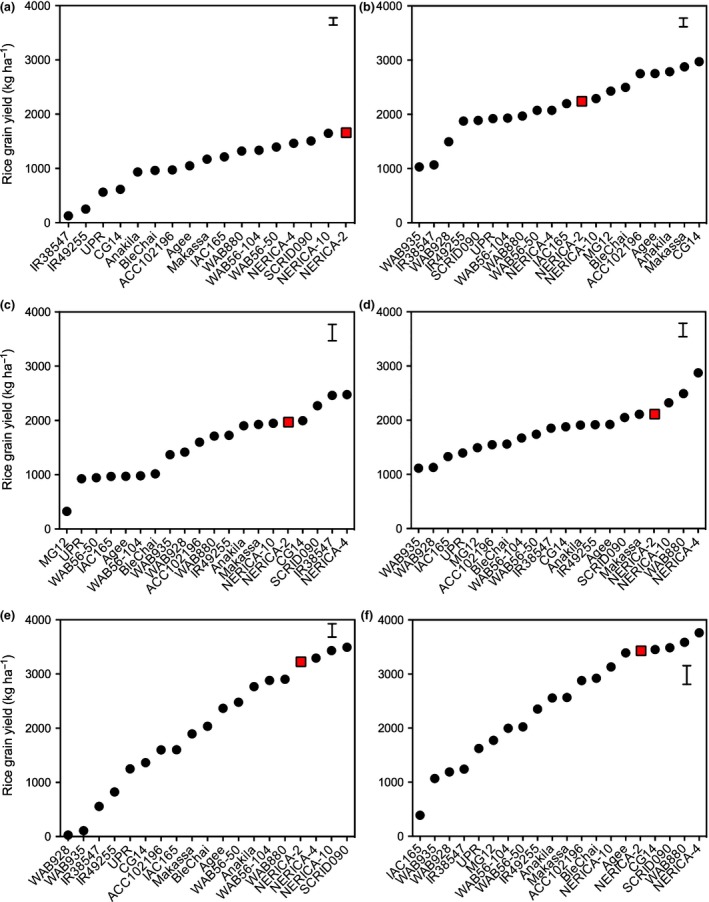
Rice grain weights under *Striga asiatica* infestation in Kyela in (a) 2014 and (b) 2015, *Striga hermonthica* infestation in Mbita, Kenya in (c) 2014 and (d) 2015 and *S. hermonthica* infestation in Namutumba, Uganda in (e) 2014 and (f) 2015. Bars represent ± SE of the least squares (LS) means. Red boxes indicate the position of resistant check genotype NERICA‐2.

Under conditions of generally low *S. asiatica* infection levels, at Kyela in 2014, the resistant check genotype NERICA‐2 had the highest rice grain yields (Fig. 5a) but yields were generally low. In 2015, the rice grain yields were much higher overall, despite the higher overall *Striga* infection levels (Fig. 5b). ACC102196, Agee, Anakila, Makassa and CG14, all *O. glaberrima*, had significantly (*P *<* *0.05) higher grain yields, while WAB935, IR38547 and WAB928 had significantly (*P *<* *0.01) lower yields than check genotype NERICA‐2.

In Mbita, rice grain yields under *S. hermonthica*‐infested conditions were comparable across years (Fig. 5c,d). A large number of genotypes (12 in 2014 and 13 in 2015) were statistically as high yielding as NERICA‐2 and only one genotype (NERICA‐4 in 2015) had a significantly (*P *<* *0.05) higher yield than NERICA‐2. The remaining seven (in 2014) and five (in 2015) had significantly (*P *<* *0.05) lower yields than NERICA‐2.

In Namutumba, grain yields as high as that of NERICA‐2 were obtained from six genotypes in 2014 (WAB56‐104, Anakila, NERICA‐10, SCRID090, WAB880 and NERICA‐4) and eight genotypes in 2015 (ACC102196, Ble Chai, NERICA‐10, Agee, CG14, SCRID090, WAB880 and NERICA‐4) (Fig. 5d,e). All other genotypes had significantly lower grain yields. NERICA‐2 and SCRID090 were the most stable in yield across years, while a number of genotypes showed high yield variation between years. Across sites and years, NERICA‐2, ‐4 and ‐10, and to a lesser extent SCRID090 showed stable high yields when grown under *Striga*‐infested conditions.

**Figure 6:**
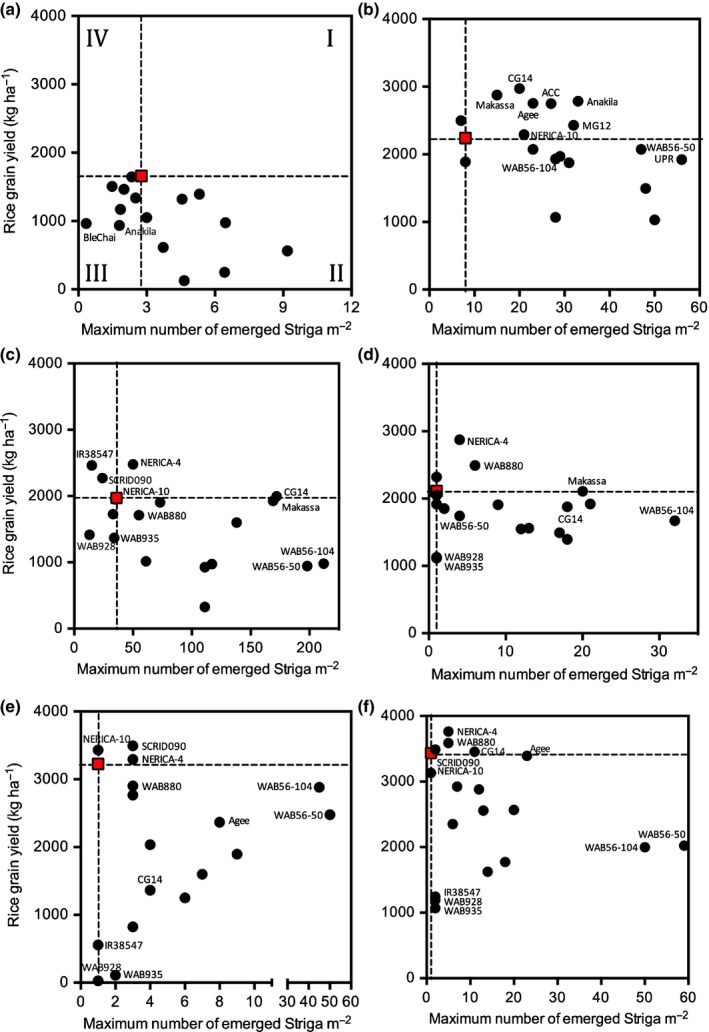
Rice grain yields plotted against maximum number of emerged *Striga* plants under *Striga asiatica* infestation in Kyela in (a) 2014 and (b) 2015, *Striga hermonthica* infestation in Mbita, Kenya in (c) 2014 and (d) 2015 and *S. hermonthica* infestation in Namutumba, Uganda in (e) 2014 and (f) 2015. Red boxes indicate the position of resistant check genotype NERICA‐2. Roman numerals in (a) refer to different quadrants with groups of genotypes relative to NERICA‐2.

In situations with generally high *Striga* infection levels (Kyela 2015 and Mbita 2014), genotype means of rice grain yields showed significant (*P *<* *0.05) negative correlations with means of maximum aboveground *Striga* numbers (*NS*_max_) (Table S3). No significant correlations were observed in other experiments. Comparing *NS*_max_ to rice grain yields, per genotype, with reference lines (horizontal and vertical) through NERICA‐2 enables the identification of genotypes in four quadrants, relative to the check genotype (Fig. 6): quadrant I comprises genotypes that are more susceptible but also higher yielding than NERICA‐2 under *Striga*‐infested conditions; quadrant II contains genotypes that are more susceptible and lower yielding than NERICA‐2 under *Striga* infestation; genotypes in quadrant III are more resistant but lower yielding than NERICA‐2 under *Striga* infestation and genotypes in quadrant IV are more resistant and higher yielding than NERICA‐2 under *Striga* infestation.

Very few genotypes were found in quadrant IV in any of the years and sites. In Kyela in 2014, there was no genotype with a better performance than NERICA‐2 and in 2015, only Ble Chai had a similar resistance level and as high a yield as NERICA‐2. In Mbita, in 2014, only IR38547 and SCRID090 performed similarly to NERICA‐2. The performance of NERICA‐10 was similar to that of NERICA‐2 in terms of *S. hermonthica* resistance and yield in both years in Mbita and in Namutumba but none of the genotypes performed better than NERICA‐2.

Seven genotypes were identified in quadrant I in Kyela in 2015. Six of them were *O. glaberrima* genotypes (Makassa, CG14, Agee, ACC102196, Anakila and MG12) and the other one was an interspecific (NERICA‐10). At the *S. hermonthica‐*infested field sites, NERICA‐4 was consistently as high or higher yielding despite higher *Striga* infection levels than NERICA‐2. Other genotypes that featured in this quadrant under *S. hermonthica* infestation were WAB880, CG14, SCRID090, Makassa and Agee. WAB56‐50 and ‐56‐104, the *O. sativa* parents of the NERICA genotypes, were almost always in quadrant II, with higher *Striga* infection levels and lower yields than NERICA‐2. Some genotypes were as resistant as or more resistant than NERICA‐2, but had much lower yields (quadrant III), notably Ble Chai and Anakila in Kyela in 2014, WAB928 and WAB935 in Mbita and WAB928, WAB935 and IR38547 in Namutumba.

### Genetic variation in *Striga* tolerance and host‐plant morphological and physiological traits depicting tolerance

To shed light on the role of tolerance in host‐plant performance, a pot experiment was conducted with *Striga*‐free compared with *Striga*‐infested plants of a subset of genotypes, with CG14, ACC102196, Makassa and NERICA‐10 as potential tolerant lines and with *S. asiatica* from Kyela as the parasite ecotype. Given the significant correlations between rice grain yield and rice plant height in the field (Table S3), we used plant height as a proxy for crop performance in the pot experiment.

Highly significant (*P *<* *0.01) negative effects of *Striga* on plant height were observed on 43‐d‐old rice plants (Table S5). There was a significant infection by genotype interaction effect on rice plant height at 43 (*P *=* *0.024) and 57 (*P *=* *0.0004) DAS as well as on the maximum plant height (*P *=* *0.0038). Significant genotypic differences were observed in *Striga*‐inflicted (maximum) height (*P *<* *0.0001; *F *=* *17.6) losses relative to uninfected control plants. Height losses across genotypes ranged from 32% (ACC102196) to 63% (IAC165).

**Figure 7:**
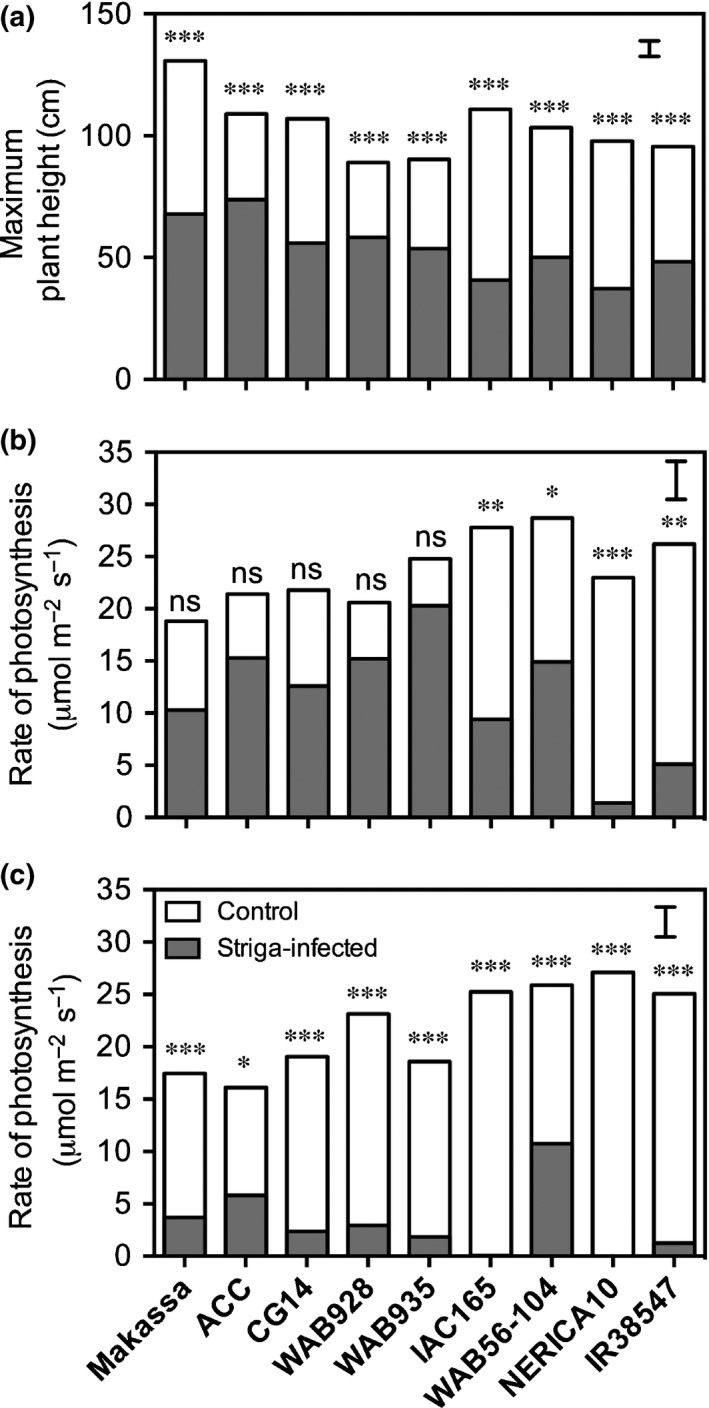
(a) Maximum rice plant height and (b, c) light‐saturated photosynthesis at (b) 30 and (c) 45 d after sowing (DAS) for a subset of genotypes grown in *Striga asiatica*‐infested (grey bars) and *Striga*‐free control (white bars) pots. Significant within‐genotype differences between infected and uninfected plants are indicated: *, *P *<* *0.05; **, *P *<* *0.01; ***, *P *<* *0.001; ns, not significant. Error bars indicate ± SE.

Significant (*P *<* *0.05) negative effects of *Striga* infection on leaf photosynthesis were observed on 30‐d‐old rice plants and even more pronounced effects were observed 15 d later (Table S5). Infection by genotype effects on leaf photosynthesis were only significant at 45 DAS. When compared within genotypes, four genotypes showed a significantly (*P *<* *0.05) lower rate of photosynthesis in leaves of *Striga*‐infected plants compared with the *Striga*‐free controls at 30 DAS (Fig. 7b). Photosynthesis of genotypes ACC102196, Makassa, CG14, WAB928 and WAB935 was not significantly affected at 30 DAS. At 45 DAS, all but one genotype (ACC102196) showed highly significantly (*P *<* *0.001) reduced photosynthesis levels in *Striga*‐infected compared with *Striga*‐free plants (Fig. 7c). In ACC102196, the reduction of photosynthesis was also significant (*P *<* *0.05) but less pronounced compared with other genotypes.

For an accurate assessment of tolerance, the genotype‐specific differences in *Striga* infection levels should be considered. In the pot experiment, CG14 (*NS*_max_ = 10) was significantly (*F *=* *15.7; *P *<* *0.0001) more resistant than six other genotypes. Only NERICA‐10 (*NS*_max_ = 15) and IR38547 (*NS*_max_ = 16) were equally resistant. With an *NS*_max_ of 43 *Striga* plants, WAB928 was significantly more susceptible than any other genotype except ACC102196 (*NS*_max_ = 31). The latter was as susceptible as IAC165, WAB935, WAB56‐104 and Makassa (*NS*_max_ = 22–27).

**Figure 8:**
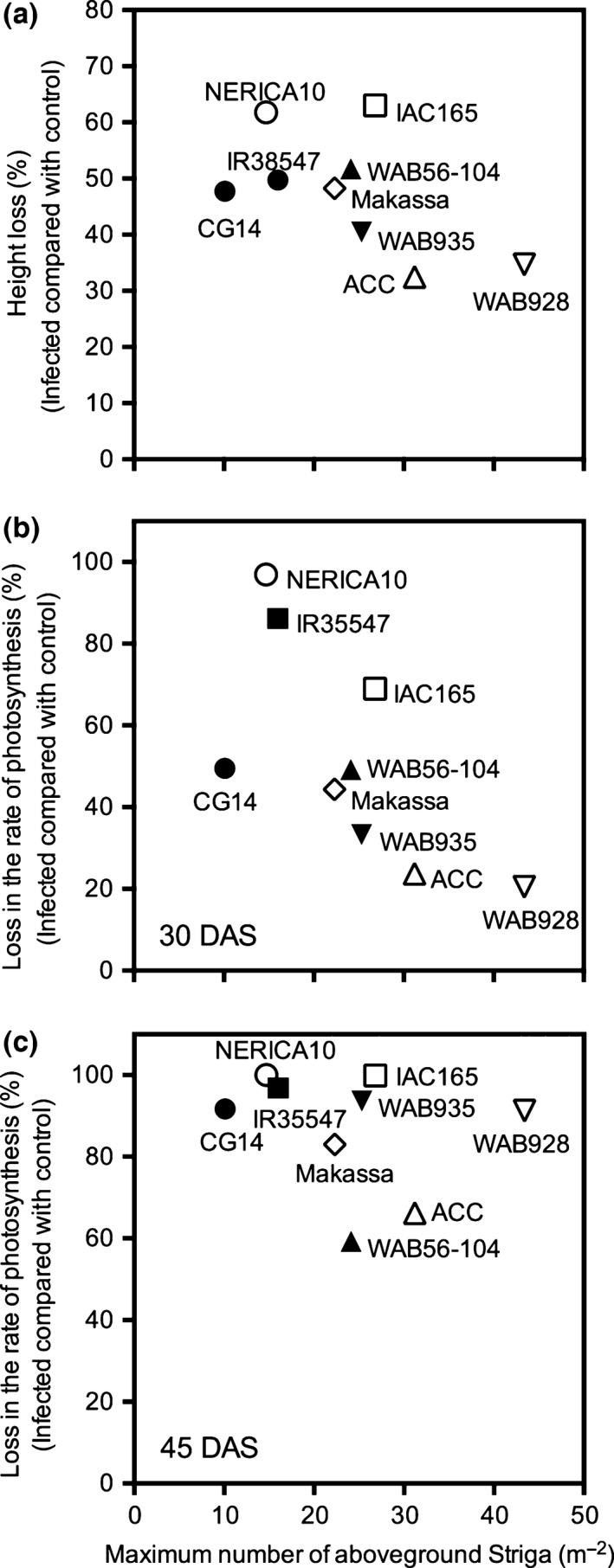
*Striga asiatica*‐inflicted losses (%) in (a) maximum rice plant height and (b, c) light‐saturated photosynthesis at (b) 30 and (c) 45 d after sowing (DAS), relative to the *Striga*‐free control plants, plotted against the maximum number of emerged *Striga* plants, for a subset of genotypes grown in the pot experiment. Genotypes are indicated by different symbols.

The extent of height losses was relatively independent of infection level (Fig. 8a). A number of genotypes with small to moderate height losses compared with controls (ACC102196, WAB928 and WAB935) had high *Striga* infection levels, while some genotypes with the greatest height losses (e.g. NERICA‐10) were among the least infected. Comparison of relative height losses between genotypes with similar infection levels indicated genotype differentiation in *Striga* effects. For NERICA‐10, *Striga* infection had a greater effect on plant height than equally resistant CG14. Given their high infection levels, ACC102196 and to a lesser extent WAB928 were less affected by *Striga* in terms of plant height reduction.

Losses in photosynthesis were also relatively independent of infection level (Fig. 8b,c). Again, the more resistant genotypes seemed to incur higher losses than the more susceptible ones, with more pronounced differences at 30 DAS (Fig. 8b) compared with 45 DAS (Fig. 8c). At 30 DAS, NERICA‐10 and IR38547 showed the greatest negative effects of *Striga*. Relatively low (≤ 50%) *Striga*‐inflicted losses in photosynthesis at 30 DAS were observed with CG14, Makassa, WAB56‐104, WAB935, ACC1021196 and WAB928. Most notable were ACC1021196 and WAB928, as they showed the smallest effects despite the highest *Striga* infection levels (Fig. 8b). Fifteen days later, at 45 DAS, *Striga* effects on photosynthesis were more severe, leading to near‐total losses in the majority of genotypes, irrespective of *Striga* infection level. Only WAB56‐104 and ACC102196 maintained these losses well below 80% (Fig. 8c).

## Discussion

### Does *Striga* resistance occur among genotypes and is it *Striga* species‐ or ecotype‐specific or broad‐spectrum?

At each site, a relatively large number of resistant rice germplasms were confirmed or newly identified. However, the level of resistance of rice genotypes in the field varied with *Striga* species and ecotype, as well as between sites and years. Climate variations affected overall *Striga* infection levels across genotypes, as shown before by Johnson *et al*. (1997). In the current study this is illustrated by the differences in overall infection levels between 2014 and 2015 at Kyela and Mbita which were associated with clear differences in rainfall between the years. This was further supported by the observation that in Namutumba, where rainfall was comparable in the two years, *Striga* infection levels were similar. However, environmental effects did not alter the expression of resistance; at low infection levels, it just became more difficult to distinguish between the resistance levels of the genotypes. In years of high infection, quantitative differences in resistance were more obvious and the resistance rankings corresponded well with those obtained in the controlled environment experiments.

The genotype rankings in the *S. hermonthica‐*infested sites at Mbita and Namutumba were similar. Consistent broad‐spectrum resistance against *S. hermonthica* (hence against both ecotypes) was observed among a large number of genotypes including NERICA‐2, ‐4 and ‐10, WAB928, ‐935 and ‐880, IR38547 and ‐49255, SCRID090, Ble Chai and Anakila. In Kyela, many genotypes resistant to *S. asiatica* were also found (i.e. Ble Chai, NERICA‐2, ‐4, and ‐10, SCRID090, CG14, Makassa and Agee). The resistance in the NERICA cultivars confirms previous findings (Cissoko *et al*., 2011; Jamil *et al*., 2011; Rodenburg *et al*., 2015; Samejima *et al*., 2016).

While resistance rankings at Namutumba and Mbita (*S. hermonthica*) were very similar, differences were observed in overall infection levels between the ecotypes at both sites. The field infection levels in Namutumba were similar and rather low in both years, while in Mbita the infection levels were similar to the levels in Namutumba in 2015 but much higher in 2014. The experiments carried out under controlled environment conditions showed similar results; higher infection levels overall were observed with *S. hermonthica* from Mbita than with *S. hermonthica* from Namutumba, suggesting that the Mbita ecotype was more virulent than the Namutumba ecotype for these rice genotypes. Between *Striga* species there were even more notable differences. First, against *S. asiatica* very few, if any, genotypes showed effective post‐attachment resistance in the rhizotron experiment, to the extent shown against the two *S. hermonthica* ecotypes where parasitic biomass approached zero on a number of genotypes. Second, while there was some overlap in the genotypes of rice with good resistance against *S. asiatica* and *S. hermonthica*, there were also some striking differences in species‐specific reaction types. For example, WAB928 and WAB935 were very resistant to both ecotypes of *S. hermonthica* but were among the most susceptible to *S. asiatica* in the field. Similarly, IR38547‐B‐B‐7‐2‐2 and IR49255‐B‐B‐5‐2 were resistant to *S. hermonthica* ecotypes but moderately susceptible to the *S. asiatica* ecotype. This observation on contrasting reaction types between *Striga* species was confirmed with WAB928 and IR38547 under controlled environment conditions.

Thus, our data show that some genotypes of rice exhibited ecotype‐specific resistance, others exhibited resistance against the two ecotypes of *S. hermonthica* (but not the ecotype of *S. asiatica*) and two genotypes (NERICA‐2 and SCRID090) showed very strong and reliable broad‐spectrum resistance across both parasite species and ecotypes. Three others (NERICA‐4 and ‐10 and Ble Chai) were also consistently among the more resistant to both *Striga* species and ecotypes. To determine whether the observed differences in the resistance of specific rice genotypes against the two *S. hermonthica* ecotypes and the *S. asiatica* ecotype reflect a *Striga* species difference requires follow‐up studies with a wider range of ecotypes screened under controlled environment conditions.

### Is resistance against *Striga* enough to maintain high rice grain yields under *Striga*‐infested conditions in different environments?

Encouragingly, the rice genotypes that exhibited good broad‐spectrum resistance were among the high‐yielding and farmer‐preferred varieties and thus could be introduced and promoted more widely in *Striga*‐prone areas. Moreover, they provide valuable additional sources for resistance breeding. Effective breeding, using marker‐assisted selection (MAS), would, however, require the identification of the genes or quantitative trait loci (QTLs) underlying the *Striga* resistance, as demonstrated by Swarbrick *et al*. (2009).

Some rice genotypes, that is, WAB935 and ‐928 in Kyela and IAC165, WAB56‐50 and WAB56‐104 in Mbita, were very susceptible and had low grain yields. Conversely, a number of rather susceptible genotypes, for example *O. glaberrima* genotypes Makassa, CG14, Agee and ACC102196, still had good grain yields despite relatively high infection levels. The yields obtained by some of the other less resistant genotypes, including Anakila, Agee and CG14 (all *O. glaberrima*), appeared more variable across years. Yield stability under *Striga*‐infested conditions seems therefore to be one of the merits of resistance, but more data are required to support such a conclusion. Yield performance of the NERICA cultivars, for instance, could also be the result of their general environmental adaptation and high yield potential (Saito *et al*., 2012; Sekiya *et al*., 2013).

Correlations between rice grain yields and *Striga* numbers (both *S. asiatica* and *S. hermonthica*) were only significant under situations of high parasite pressure. This confirms previous studies, both with rice (Rodenburg *et al*., 2015) and with sorghum (*Sorghum bicolor* (L.) Moench) (Rodenburg *et al*., 2005). The inconsistency of this correlation indicates that resistance, responsible for reduced *Striga* infection levels, is not the sole determinant of high yields under *Striga*‐infested field conditions. In years with lower *Striga* infection levels, tolerance and yield potential, rather than resistance, seem to be important, confirming previous studies by Rodenburg *et al*. (2005, 2015).

### Does genetic variation in tolerance to *Striga* exist in rice germplasm and which host‐plant morphological or physiological traits can be used to predict tolerance?

In studies to identify tolerance in maize (Pierce *et al*., 2003) and sorghum (Bebawi & Farah, 1981; Showemimo, 2003), the extent of stunting of the host plant is often used as an indicator for tolerance. In our study, the usefulness of the reduction in height of the main stem of *Striga*‐infected rice (as a percentage of the uninfected plant) could be assessed as this parameter ranged between *c*. 30% and 65% as a function of the rice genotype. Based on this measure, ACC102196 and WAB928 proved to be more tolerant than the other cultivars. This is in agreement with the yield data from the field trials for ACC101196, but not for WAB928 (which was very low yielding). Possibly the yield potential or environmental adaptation of WAB928 is suboptimal, causing a low baseline yield level, but this requires additional investigation.

In this study, measurement of the rate of photosynthesis at 30 DAS was a better discriminator of tolerance (lower levels of *Striga* damage) between the rice genotypes than the reduction in height of infected plants. The ability of maize and sorghum varieties to maintain high rates of photosynthesis under *Striga* infection has also proved a good indicator for physiological tolerance (Gurney *et al*., 2002; Rodenburg *et al*., 2008). The rice cultivars began to exhibit the damaging effects of *Striga* very quickly after attachment, confirming previous findings (e.g. Cechin & Press, 1994; Watling & Press, 2001). Measurements of the rate of photosynthesis also became less discriminating and predictive of tolerance with time after infection, illustrating the need to make these measurements early during the host–parasite interaction.

The *O. glaberrima* genotypes Makassa, ACC102196 and CG14 showed good tolerance in comparison to many of the *O. sativa* genotypes used in this study. They showed no significant *Striga*‐induced reductions in leaf photosynthesis at early stages of the host–parasite interaction when CO_2_ assimilation rates of other genotypes were already severely reduced. CG14 and Makassa were also relatively high yielding in Mbita, even in a year when infection levels were generally high such as 2014. All the *O. glaberrima* genotypes screened at Kyela showed higher yields at higher *S. asiatica* infection levels than NERICA‐2. As the species *O. glaberrima* is generally not high yielding (Dingkuhn *et al*., 1998), these relatively high yields, despite high infection levels, are probably indeed the outcome of effective physiological tolerance. This observation also agrees with that of Johnson *et al*. (1997), who found lower levels of *Striga* damage on *Striga*‐infected *O. glaberrima* cultivars compared with *O. sativa* cultivars. Based on these observations, *O. glaberrima* germplasm may be a good source of ‘tolerance’ genes that could be exploited for breeding this trait into *Striga*‐resistant cultivars.

Resistance and tolerance are not often found together in the same genotype. Some susceptible cultivars show high levels of tolerance to *Striga* damage, for example ACC102196 and WAB928 in the current study, while some cultivars with good resistance are highly sensitive to one or two parasite attachments, for example NERICA‐10. A similar combination of high resistance but high sensitivity was observed in the sorghum genotype N13 (Rodenburg *et al*., 2005). Thus, in order to control *Striga* and maintain high yields, both tolerance and resistance are required in cultivars recommended to farmers. The high genetic variability of the parasite seed bank means that even strongly resistant cultivars may be infected by a few *Striga* individuals, leading to yield losses if the genotypes do not possess some degree of tolerance. Conversely, tolerant genotypes will allow the build‐up of the *Striga* seed bank if they do not possess some degree of resistance. Thus, varieties with both resistance and tolerance, grown in combination with other control measures, will provide a feasible and durable solution to farmers and delay the evolution of virulence in parasite populations.

### Conclusions

This is the first study to compare the resistance levels of the same suite of rice genotypes in three regions of Africa infested by different genetic ecotypes of *S. hermonthica* and *S. asiatica*. First, we have shown that the resistance ranking of rice genotypes in the field was very similar to that under controlled environment conditions, thus demonstrating that resistance was genetically determined. Second, some rice genotypes exhibited broad‐spectrum resistance to all the ecotypes of *Striga*, while others exhibited ecotype‐specific resistance. Finally, the resistance rankings of rice genotypes at Mbita and Namutumba – both areas infested with *S. hermonthica* ecotypes – were similar, suggesting that the parasite virulence genes in these populations were similar. This contrasted with the virulence profile of the *S. asiatica* ecotype, as some of the rice genotypes exhibited different resistance rankings. We have shown that tolerance was also genetically determined, and the level of tolerance varied across genotypes, as evident from the extent to which *Striga*‐inflicted losses in plant height and photosynthesis were differentially mitigated across genotypes, but it was independent of the level of resistance in these genotypes. Thus, the grain yield of a given rice genotype obtained in a *Striga*‐infested field is the result of the inherent yield potential of that genotype and the level of host resistance and tolerance against the field‐specific parasite species and ecotype. These novel findings provide invaluable information for molecular and conventional rice breeders, and strongly support the need for predictive breeding strategies to be employed for affected staple crops such as rice. For such a predictive breeding approach, knowledge of the molecular genetic background of host resistance and tolerance can be coupled to that of the prevailing parasite ecotype in a specific region in order to breed cultivars with effective defence.

## Author contributions

J.R., M.C., J.B., C.A.O.M., and J.D.S. planned and designed the research; J.R., M.C., N.K., R.I. and I.M. performed experiments and collected data; I.D. analysed data; J.R. M.C., C.A.O.M., I.D. and J.D.S. wrote the manuscript.

## Supporting information

Please note: Wiley Blackwell are not responsible for the content or functionality of any Supporting Information supplied by the authors. Any queries (other than missing material) should be directed to the *New Phytologist* Central Office.

**Table S1** Overview of experimental conditions of the field trials conducted at Kyela, Tanzania, Namutumba, Uganda and Mbita, Kenya (2014 and 2015)
**Table S2** ANOVA output on maximum emerged *Striga* numbers observed in the field at three sites (Kyela, Mbita and Namutumba) in the years 2014 and 2015 with 20 rice genotypes
**Table S3** Spearman rank correlations between LS‐Means of maximum aboveground *Striga* numbers and aboveground *Striga* biomass dry weights at harvest, between *NS*_max_ and rice grain yields, and between rice grain yields and rice plant height for the field data in Kyela, Mbita and Namutumba in both seasons (2014 and 2015)
**Table S4** ANOVA output on rice grain yields and rice straw dry weights observed in the field at three sites (Kyela, Mbita and Namutumba) in the years 2014 and 2015 with 20 rice genotypes
**Table S5** ANOVA output on maximum rice plant height, and plant height at 43 and 57 d after sowing (DAS), and photosynthesis at 30 and 45 DAS, with *Striga* infection and rice genotype as sources of variation
