# Supplementary material for: Genetic variation and host–parasite specificity of Striga resistance and tolerance in rice: the need for predictive breeding
Source: New Phytol. 2017 Feb 13;214(3):1267–80. doi: 10.1111/nph.14451 (PMC5412873; doi:10.1111/nph.14451)
Supplement: Supplementary file 1 — Table S1 Overview of experimental conditions of the field trials conducted at Kyela, Tanzania, Namutumba, Uganda and Mbita, Kenya (2014 and 2015) Table S2 ANOVA output on maximum emerged Striga numbers observed in the field at three sites (Kyela, Mbita and Namutumba) in the years 2014 and 2015 with 20 rice genotypes Table S3 Spearman rank correlations between LS‐Means of maximum aboveground Striga numbers and aboveground Striga biomass dry weights at harvest, between NS max and rice grain yields, and between rice grain yields and rice plant height for the field data in Kyela, Mbita and Namutumba in both seasons (2014 and 2015) Table S4 ANOVA output on rice grain yields and rice straw dry weights observed in the field at three sites (Kyela, Mbita and Namutumba) in the years 2014 and 2015 with 20 rice genotypes Table S5 ANOVA output on maximum rice plant height, and plant height at 43 and 57 d after sowing (DAS), and photosynthesis at 30 and 45 DAS, with Striga infection and rice genotype as sources of variation [file NPH-214-1267-s001.pdf]

New Phytologist

**_New Phytologist_ Supporting Information Tables S1–S5**

Article title: Genetic variation and host–parasite specificity of Striga resistance and tolerance in rice: the need for predictive breeding

Authors: Jonne Rodenburg, Mamadou Cissoko, Nicholas Kayongo, Ibnou Dieng, Jenipher Bisikwa, Runyambo Irakiza, Isaac Masoka, Charles A. O. Midega and Julie D. Scholes

Article acceptance date: 15 December 2016

The following Supporting Information is available for this article:

**Table S1** Overview of experimental conditions of the field trials conducted at Kyela, Tanzania, Namutumba, Uganda and at Mbita, Kenya (2014 and 2015)

**Table S2** ANOVA output on maximum emerged Striga numbers observed in the field at three sites (Kyela, Mbita and Namutumba) in years 2014 and 2015 with 20 rice genotypes

**Table S3** Spearman rank correlations between LS-Means of maximum aboveground Striga numbers and aboveground Striga biomass dry weights at harvest, between $NS_{max}$ and rice grain yields, and between rice grain yields and rice plant height for the field data in Kyela, Mbita and Namutumba in both seasons (2014, 2015)

**Table S4** ANOVA output on rice grain yields and rice straw dry weights observed in the field at three sites (Kyela, Mbita and Namutumba) in years 2014 and 2015 with 20 rice genotypes

**Table S5** ANOVA output on maximum rice plant height, and plant height at 43 and 57 d after sowing (DAS), and photosynthesis at 30 and 45 DAS, with Striga infection and rice genotype as sources of variation

**Table S1** Overview of experimental conditions of the field trials conducted at Kyela, Tanzania, Namutumba, Uganda and at Mbita, Kenya (2014 and 2015)

| | **Kyela – Tanzania (*S.*** | | **Namutumba – Uganda (*S.*** | | **Mbita – Kenya (*S.*** | |
|---|---|---|---|---|---|---|
| Geo-coordinate | 9°37’ S - 33°52’ E | | 00° 51’ N, 33°41’ E | | 00°43’ S - 34°20’ E | |
| Altitude (m asl) | 525 | | 1,125 | | 1,141 | |
| Season | Feb./March-July | | March - August | | March-September | |
| Sowing dates 2014/2015 | 5 March 2014 /25 February 2015 | | 13 March 2014/12 March 2015 | | 18 March 2014/17 March 2015 | |
| Cumulative Rainfall | 2,136 | 966 | 720 | 876 | 452 | 685 |
| Genotypes | 20 | | 20 | | 20 | |
| Net plot size ($m^2$) | 93.8 $m^2$ | | 68.8 $m^2$ | | 68.8 $m^2$ | |
| Net subplot size ($m^2$) | 4.69 $m^2$ | | 3.44 $m^2$ | | 3.44 $m^2$ | |
| Fertilizer application | 100 kg $ha^{-1}$ N-P-K : 20-10- | | 50 kg $ha^{-1}$ N-P-K : 17-17-17 | | 50 kg $ha^{-1}$ N-P-K : 17-17- | |
| Striga infestation density | 0.21 g | | 0.29 g | 0.26 g | 0.29 g | |
| Soil fertility parameters | ***2014*** | ***2015*** | ***2014*** | ***2015*** | ***2014*** | ***2015*** |
| pH | 5.75 | 4.96 | 6.06 | 6.03 | 7.85 | 7.83 |
| N (%) | 0.16 | 0.10 | 0.06 | 0.10 | 0.14 | 0.11 |
| P (ppm) | 9.68 | 9.80 | 7.41 | 6.72 | 12.72 | 12.51 |
| K (ppm) | 317 | 304 | 135 | 174 | 353 | 416 |
| Sand : silt : clay | 63:13:25 | | 68:12:20 | | | |

*Seed weight of *S. asiatica*: 0.0000037 g; seed weight of *S. hermonthica*: 0.0000071 g (according to Parker & Riches, 1993).

**Table S2** ANOVA output on maximum emerged Striga numbers (*NSmax*) observed in the field at three sites (Kyela, Mbita and Namutumba) in year (Y) 2014 and 2015 with 20 rice genotypes (G)

| | | **Kyela** | | | **Mbita** | | | **Namutumba** | | |
|---|---|---|---|---|---|---|---|---|---|---|
| *Year* | *Source* | df | *F** | *P* | df | *F* | *P* | df | *F* | *P* |
| *2014 & 2015* | Y | 1 | 59.8 | <0.0001 | 1 | 44.8 | <0.000 | 1 | 6.7 | 0.0275 |
| | G | 19 | 17.7 | <0.0001 | 19 | 73.0 | <0.000 | 19 | 108.9 | 0.0000 |
| | Y × G | 19 | 3.0 | 0.0002 | 19 | 9.6 | <0.000 | 19 | 2.7 | 0.0007 |
| *2014* | G | 19 | 8.5 | <0.0001 | 19 | 159.0 | <0.000 | 19 | 61.2 | <0.0001 |
| *2015* | G | 19 | 26.3 | <0.0001 | 19 | 28.5 | <0.000 | 19 | 62.2 | <0.0001 |

*For all analyses on *NSmax* a generalized linear mixed model (McCullagh & Nelder, 1989) was used under the assumption of a Poisson distribution.

**Table S3** Spearman rank correlations (*r* = correlation coefficient) between LS-means of maximum aboveground Striga numbers (*NSmax*) and aboveground Striga biomass dry weights at harvest (*StrigaDW*), between *NSmax* and rice grain yields (*Yield*), and between rice grain yields and rice plant height (*Height*) for the field data in Kyela, Mbita and Namutumba in both seasons (2014, 2015)

| **Parameters** | **Year** | **Kyela** | | **Mbita** | | **Namutumba** | |
|---|---|---|---|---|---|---|---|
| | | *r* | *P* | *r* | *P* | *r* | *P* |
| *NSmax – StrigaDW* | 2014 | 0.902 | <0.0001*** | 0.880 | <0.0001*** | 0.837 | <0.0001*** |
| | 2015 | 0.670 | 0.0288* | 0.921 | <0.0001*** | 0.838 | <0.0001*** |
| *NSmax – Yield* | 2014 | -0.289 | 0.216 | -0.486 | 0.0299* | -0.030 | 0.904 |
| | 2015 | -0.477 | 0.0336* | -0.213 | 0.367 | -0.290 | 0.215 |
| *Yield – Height* | 2014 | 0.494 | 0.027* | 0.152 | 0.523 | 0.536 | 0.018* |
| | 2015 | 0.702 | 0.0006*** | 0.331 | 0.154 | 0.615 | 0.0039** |

*, significant at the 95% confidence level; **, significant at the 99% confidence level; ***, significant at the 99.9% confidence level.

New Phytologist

**Table S4** ANOVA output on rice grain yields and rice straw dry weights observed in the field at three sites (Kyela, Mbita and Namutumba) in year (Y) 2014 and 2015 with 20 rice genotypes (G)

| | | | **Kyela** | | | **Mbita** | | | **Namutumba** | | |
|---|---|---|---|---|---|---|---|---|---|---|---|
| | *Year* | *Source* | df | *F** | *P* | df | *F* | *P* | df | *F* | *P* |
| *Grain yield* | *2014 & 2015* | Y | 1 | 183.1 | <0.0001 | 1 | 1.7 | 0.2209 | 1 | 4.3 | 0.0649 |
| | | G | 19 | 23.9 | <0.0001 | 19 | 7.4 | <0.0001 | 19 | 26.4 | <0.0001 |
| | | Y × G | 19 | 10.5 | <0.0001 | 19 | 1.8 | 0.0319 | 19 | 4.8 | <0.0001 |
| | *2014* | G | 19 | 39.5 | <0.0001 | 19 | 4.7 | <0.0001 | 19 | 22.1 | <0.0001 |
| | *2015* | G | 19 | 10.7 | <0.0001 | 19 | 4.8 | <0.0001 | 19 | 11.7 | <0.0001 |
| *Straw dry weight* | *2014 & 2015* | Y | 1 | 188.0 | <0.0001 | 1 | 5.6 | 0.0389 | 1 | 0.1 | 0.8237 |
| | | G | 19 | 12.2 | <0.0001 | 19 | 20.9 | <0.0001 | 19 | 21.1 | <0.0001 |
| | | Y × G | 19 | 12.8 | <0.0001 | 19 | 1.8 | 0.0298 | 19 | 2.9 | 0.0005 |
| | *2014* | G | 19 | 12.5 | <0.0001 | 19 | 7.3 | <0.0001 | 19 | 8.7 | <0.0001 |
| | *2015* | G | 19 | 12.8 | <0.0001 | 19 | 16.6 | <0.0001 | 19 | 15.5 | <0.0001 |

*Analyses were done following a linear mixed model.

**Table S5** ANOVA output on maximum rice plant height, and plant height at 43 and 57 d after sowing (DAS), and photosynthesis at 30 and 45 DAS, with Striga infection (S) and rice genotype (G) as sources of variation

| | | **Plant height** | | | | | | **Photosynthesis** | | | |
|---|---|---|---|---|---|---|---|---|---|---|---|
| | | 43 DAS | | 57 DAS | | Maximum | | 30 DAS | | 45 DAS | |
| Source | df | *F* | *P* | *F* | *P* | *F* | *P* | *F* | *P* | *F* | *P* |
| S | 1 | 40.7 | 0.0007 | 242.5 | 0.0000 | 169.09 | <0.001 | 8.61 | 0.03 | 45.26 | <0.001 |
| G | 8 | 4.2 | 0.0008 | 4.8 | 0.0002 | 7.79 | <0.001 | 1.88 | 0.08 | 2.31 | 0.03 |
| S × G | 8 | 2.5 | 0.0240 | 4.6 | 0.0004 | 3.38 | 0.004 | 2.03 | 0.06 | 2.97 | 0.01 |
